# British Society of Echocardiography guideline for the transthoracic echocardiographic assessment of adult patients with obstructive hypertrophic cardiomyopathy receiving myosin-inhibitor therapy

**DOI:** 10.1186/s44156-025-00078-z

**Published:** 2025-06-02

**Authors:** Clare M. Culshaw, Daniel Augustine, Caroline J. Coats, Ivo Andrade, Keith Pearce, Antonis Pantazis, William Bradlow, Lauren Turvey, William Moody, Lynne Williams, Rachel Bastianen, Jane Draper, David L. Oxborough, Robert M. Cooper

**Affiliations:** 1 Liverpool Heart and Chest Hospital, Thomas Drive, Liverpool, L14 3PE UK; 2 Royal United Hospitals Bath NHS Foundation Trust, Bath, Bath, UK; 3 University of Bath, Bath, UK; 4 Queen Elizabeth University Hospital, Glasgow, UK; 5 Manchester University NHS Foundation Trust, Manchester, UK; 6 Royal Brompton and Harefield Hospital, London, UK; 7 University Hospital Birmingham, Birmingham, UK; 8 Queen Elizabeth Hospital Birmingham, Birmingham, UK; 9 Royal Papworth Hospital, London, UK; 10 Guys and St Thomas Hospital, London, UK; 11 Sports and Exercise Sciences, Liverpool John Moores University, Liverpool, UK

**Keywords:** Mavacamten, Myosin-inhibitor, Obstructive HCM, Surveillance Echocardiogram

## Introduction

Hypertrophic cardiomyopathy (HCM) is a disease characterised by myocardial hypertrophy. This is linked to dysfunction of the cardiac sarcomere that results in excessive cardiac myosin–actin cross-bridging and increased sensitivity to calcium. Mavacamten is a selective, reversible myosin-inhibitor licensed in the United Kingdom as an oral therapy to treat symptomatic obstructive Hypertrophic Cardiomyopathy (oHCM) [1, 2].

Seminal studies of Mavacamten highlighted potential risk of heart failure due to left ventricular (LV) systolic dysfunction in a small percentage of oHCM patients. Due to this risk, safety monitoring is required when integrating this therapy into clinical practice [1]. Echocardiography is a key diagnostic imaging tool when diagnosing and managing oHCM and is part of the surveillance for those patients being treated with Mavacamten. This guideline from the British Society of Echocardiography outlines a protocol for minimum standards for surveillance of patients undergoing treatment using Mavacamten.

## Background

Hypertrophic Cardiomyopathy is a disease of cardiac myocytes characterised by left ventricular hypertrophy (LVH) in the absence of abnormal loading conditions. Most commonly observed pathogenic genetic variants in those with HCM include changes in myosin heavy chain 7 (MYH7) and myosin binding protein C3 (MYBPC3) genes. These variants contribute to the excessive myosin-actin cross bridging that underpins the clinical features of the disease [3]. Half of individuals carrying a pathogenic variant express the disease by the third decade of life [3]. Additional core pathophysiological features include diastolic dysfunction, myocardial fibrosis and microvascular ischaemia. The classic form of LVH affects the basal ventricular septum although other segments of the left ventricle can also be affected.

The prevalence of HCM is estimated at 1:500 [4], with UK Biobank data suggesting a general population prevalence of LVH ≥ 15 mm in 0.11% in previously undiagnosed individuals [5]. With the improved diagnostic yield of tests, family screening and the availability of genetic testing the genetic prevalence is expected to be as high as 1:250 [6, 7]. These disease-causing genetic alterations affect the structure and function of sarcomeric proteins resulting in molecular changes that cause excessive cardiac myosin–actin cross-bridging. This leads to a rise in force generation and subsequent myocardial hypercontractility [8]. A third of patients diagnosed with HCM have evidence of LV outflow tract (LVOT) obstruction at rest by the third and fourth decades of life [3]. Another third develop evidence of LVOT obstruction with exercise [9]. This is a result of the combination of the hypertrophied basal ventricular septum encroaching the LVOT, myocardial hypercontractility, and systolic anterior motion of the mitral valve into the LVOT.

Patients predominantly present with symptoms of shortness of breath, although exertional chest pain, dizziness and/or syncope are also reported. An abnormal resting LVOT pressure gradient is defined at > 30 mmHg [2], however, a LVOT peak pressure gradient of > 50 mmHg is used as threshold for initiating treatment with Mavacamten or alternative invasive options such as alcohol septal ablation or surgical myectomy [2]. The presence and magnitude of the LVOT obstruction is a predictor of disease progression to heart failure and mortality [10] and therefore an important indicator in this patient population.

Treatment is primarily aimed at improving quality of life in those with restricting symptoms. Treating clinicians should ensure that phenocopies of HCM that required alternative treatment modalities are ruled out [2]. Historically management of patients with symptomatic LVOT obstruction includes lifestyle advice, medications with negative inotropic effect and/or invasive septal reduction therapy. Lifestyle changes include weight reduction, avoidance of dehydration and avoidance of excessive alcohol consumption. The European Society of Cardiology recommends betablockers as first line therapy, with non- dihydropyridine calcium channel blockers as a second line therapy if betablockers are either ineffective, poorly tolerated, or contraindicated. The next management step includes either Disopyramide or Mavacamten [2]. Septal reduction therapy with either alcohol septal ablation or surgical myectomy is reserved for patients with significant symptomatic LVOT obstruction refractory to medical therapy [11].

Mavacamten is a selective and reversible cardiac myosin ATPase inhibitor. It reduces the number of myosin heads in an active state. This shifts the overall myosin population towards an energy sparing, super-relaxed state, moving away from excessive cardiac myosin–actin cross-bridging. Mavacamten is reported to significantly reduce LVOT gradients, improve patients’ symptoms, improve exercise capacity, and reduce serum levels of N-terminal pro-type natriuretic peptide (NT pro BNP) and high sensitivity troponins [12, 13].

Whilst Mavacamten has been shown to improve symptoms in study populations along with reduction in LVOT obstruction, data from EXPLORER-HCM and VALOR-HCM have shown that for up to 5% of patients there will be a reduction of LV ejection fraction (LVEF) to < 50% [12, 13]. Follow up data suggested that the drop in LVEF resolved with the cessation or dose reduction of Mavacamten [12]. The MAVA-LTE study reported long term outcome up to 3 years showing sustained improvements in gradients and symptoms, with low volume of transient, reversible reduction in EF [14].

**Fig. 1:**
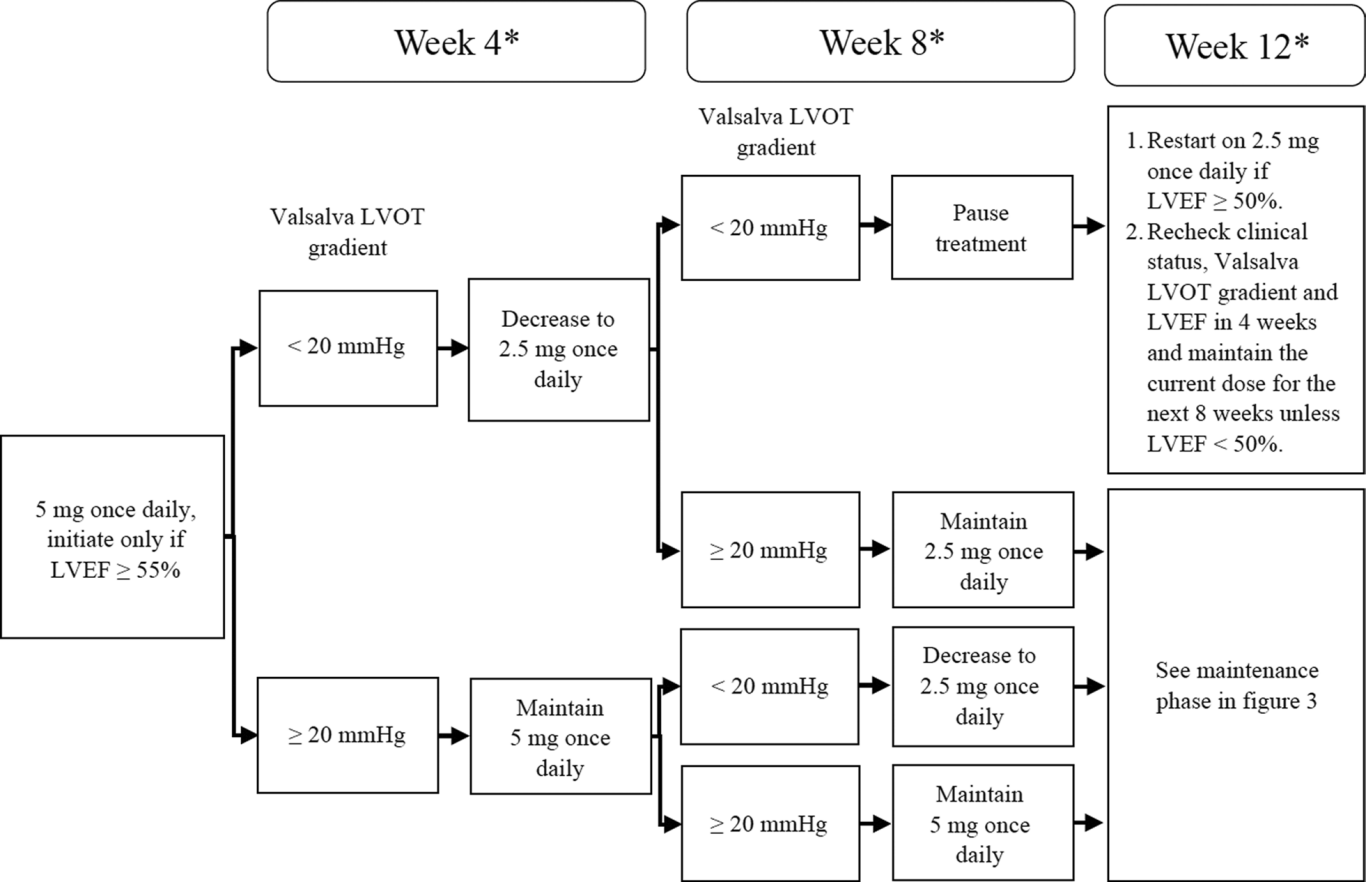
Recommended algorithm for patients who are normal and rapid CYP metabolisers. *Interrupt treatment at any point if LVEF <50%

**Fig. 2:**
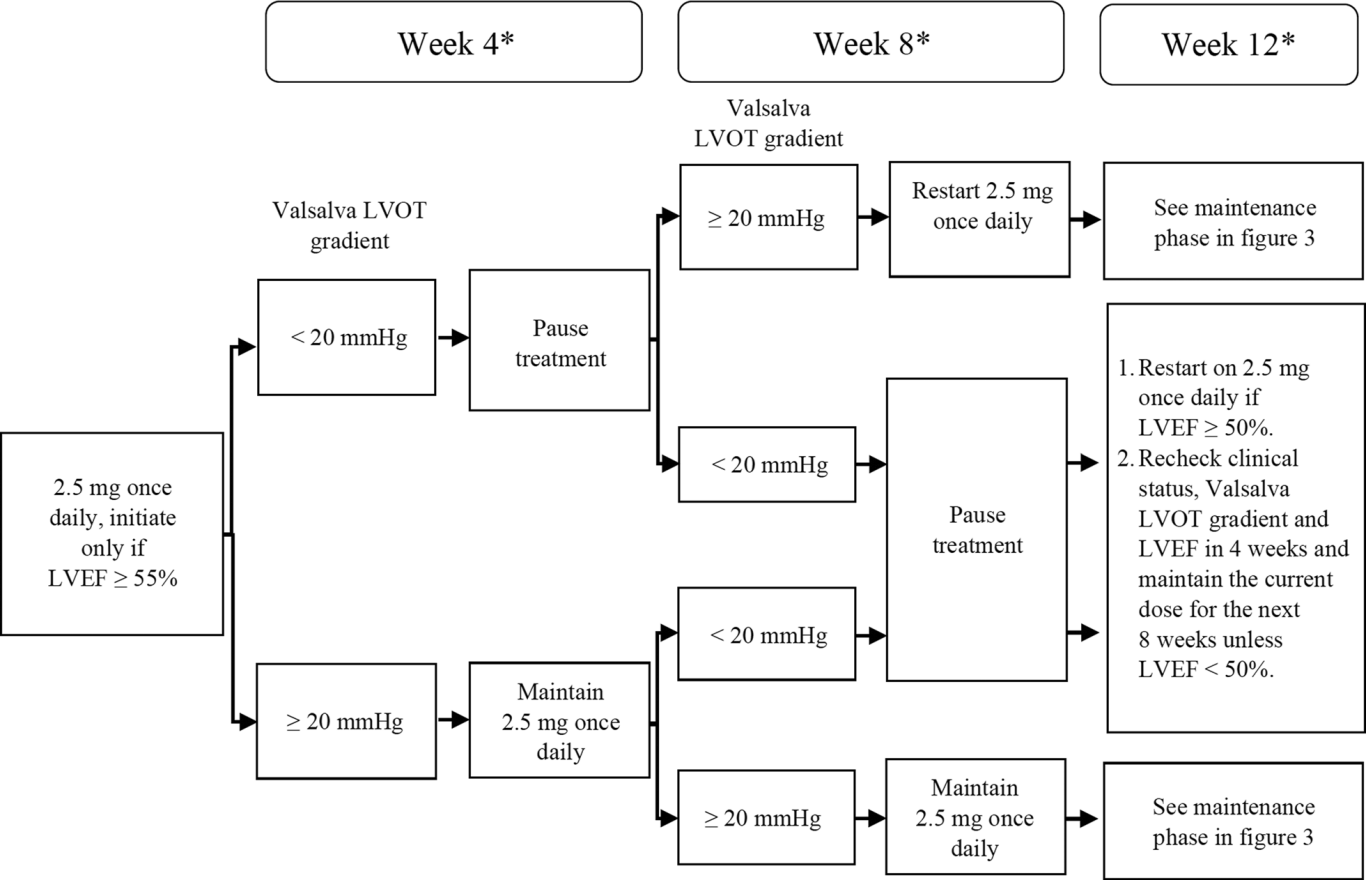
Recommended algorithm for patients who are slow/poor CYP metabolisers or while CYP status is pending. *Interrupt treatment at any point if LVEF <50%

Due to the potential risk of heart failure linked to drop in LVEF, recent National Institute for Clinical Excellence (NICE) guidance recommends a minimal level of safety monitoring that should be implemented where Mavacamten is used in clinical practice [1]. Echocardiography has been recommended as the diagnostic tool for the safety monitoring for patients prescribed Mavacamten. NICE guidance recommends surveillance echocardiography as per the summary of product characteristics of the medication at weeks 4, 8 and 12 post initiation of Mavacamten followed by 24 weekly echocardiograms long term. Echocardiography is also mandated 4 weeks after any dose change (see Figs. 1, 2). All echocardiography data will be taken in to account at a clinical review in combination with symptoms when considering dose alterations.

Due to the high frequency of surveillance echocardiograms stipulated by NICE this document by the BSE aims to standardise echocardiographic practice in this domain and support services in using clinically targeted echocardiograms to satisfy the requirements and allow safe monitoring for patients using Mavacamten. Mavacamten is currently the only licensed myosin inhibitor medication in the UK. There is the potential for other myosin inhibitor medications to reach the clinical space in the future. This document focusses on the use of Mavacamten as this was the focus of NICE technology appraisal guidance [1]. However, elements of this BSE protocol could be used or adapted to other myosin inhibitors if their use requires monitoring in a similar manner to Mavacamten.

## The role of echocardiography and recommended Mavacamten protocol

A full comprehensive echocardiographic assessment in accordance with the British Society of Echocardiography Hypertrophic Cardiomyopathy guideline [15, 16] should be undertaken before initiation of treatment with Mavacamten. This should be performed with the patient on standard medical treatment and will inform the next stage of treatment. In addition to a baseline echocardiogram, it is recommended that patients undergo pharmacogenomic testing in order to assess metabolic activity linked to removal of Mavacamten. (see Appendix 1 for pharmacogenomic guidance).

**Fig. 3:**
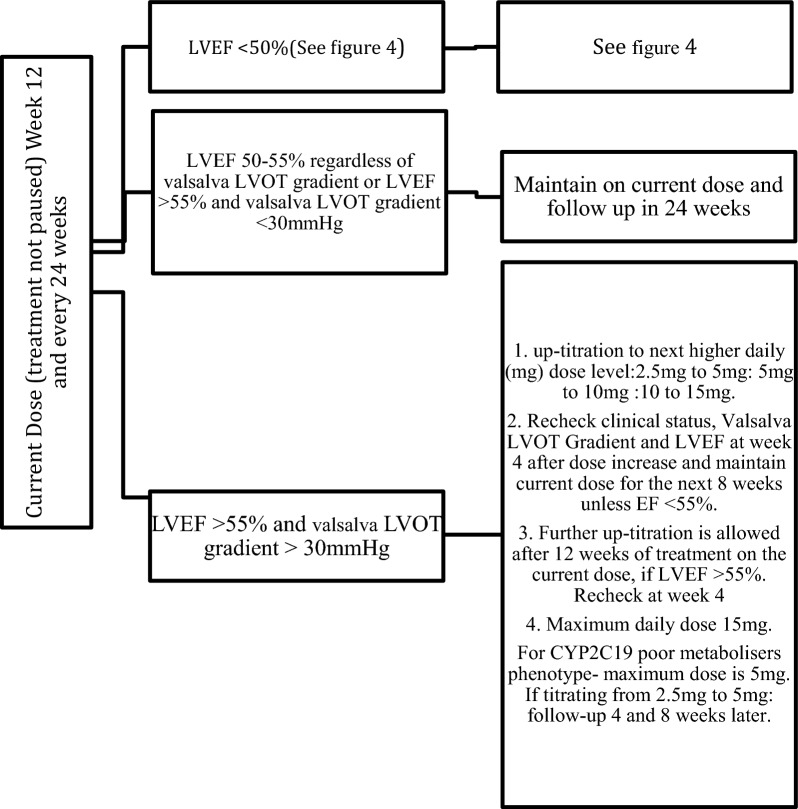
Maintenance assessment algorithm. Echocardiography should be performed every 24 weeks unless a dose titration is needed or LVEF drops to < 50%

**Fig. 4:**
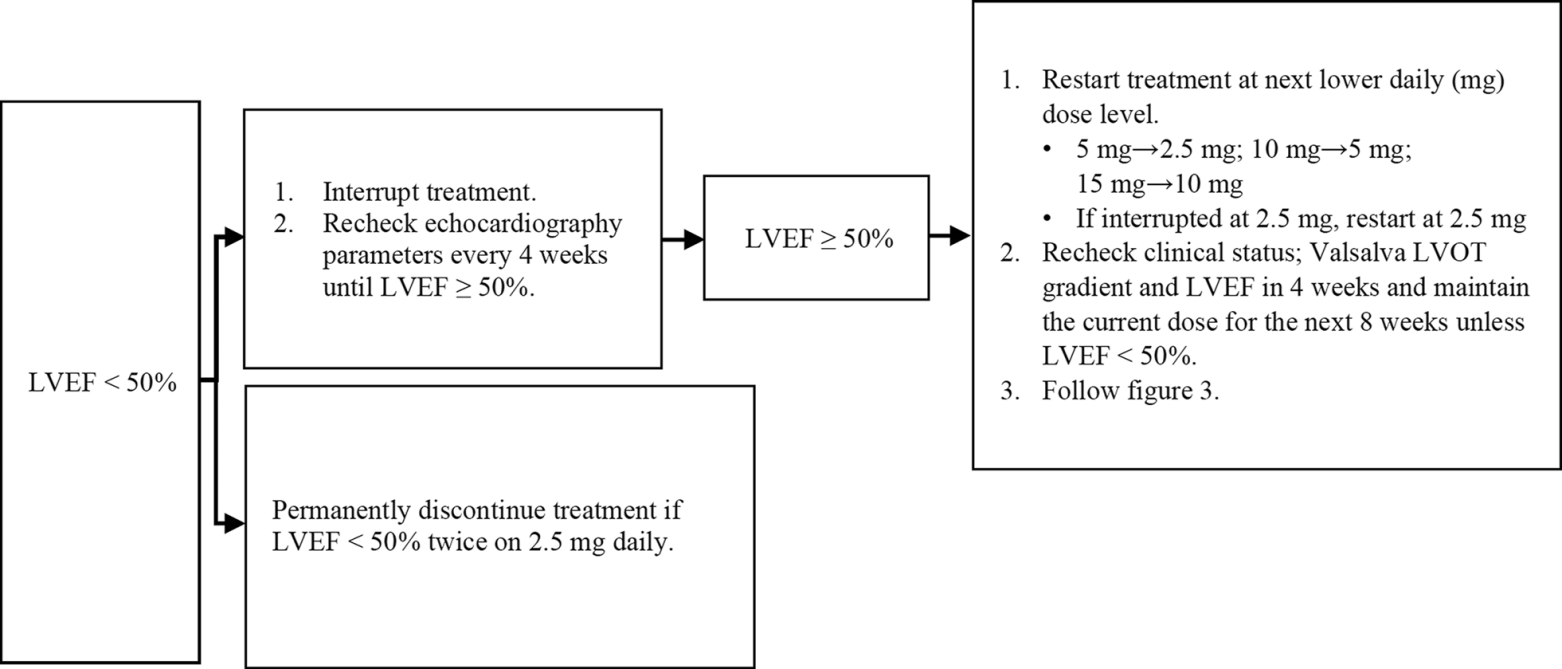
Actions if LVEF drops to < 50%. If at any visit the patient’s LVEF is < 50%, the treatment should be interrupted for at least 4 weeks and only restarted if LVEF ≥ 50%

Several practical considerations are important when evaluating echocardiographic protocols for those on myosin inhibitors:

- The standardisation of manoeuvres used in the baseline assessment of level of LVOT obstruction detected.
- The increase in volume of echocardiograms due to the high frequency of surveillance scans (see Figs. 1, 2, 3 and 4).
- The standardisation of monitoring scans.

The detection of LVOT obstruction can be achieved with provocation manoeuvres when not obvious at rest, aiming to alter the loading conditions and mimic the physiological circumstances under which LVOT obstruction is maximised. LVOT gradients should be assessed at rest and with Valsalva manoeuvre in the left lateral recumbent position. The CW doppler should be aligned with the direction of forward flow and guided by colour flow Doppler [15, 16]. It can be helpful to sweep the angle of interrogation between the LVOT and left atrium (LA) to allow for improved discrimination of LVOT gradients from mitral regurgitation Doppler profiles.

Whilst the baseline echocardiography study is expected to adhere to the full BSE protocol for Hypertrophic Cardiomyopathy [15, 16], the surveillance scans recommended by NICE guidance supports the concept of targeted echocardiography studies. For the purpose of consistency, the protocol of surveillance echocardiography should be reproducible, accurately recorded and reported. The minimum dataset for a surveillance echocardiogram should include the licensing requirements for Mavacamten as they are described in NICE Guidance [1]. There are also some echocardiographic parameters that can optionally be acquired depending on specific local departmental requirements.

## Minimum dataset

### Transthoracic echocardiography- for treatment using cardiac myosin inhibitor for treatment of obstructive hypertrophic cardiomyopathy

**Table:** 

Measurement	View	Modality	Explanation	Image
Visual assessment	PLAX view	2D	Parasternal long axis (PLAX) is optimised to demonstrate the best image available [15, 16]	
Visual assessment of MR	plax view	2D and CF doppler	Optimise colour flow doppler (CFD) settings. Adjust the lateral CFD region of interest (ROI) to include 1 cm of the LV on the left lateral border and the roof of the LA on the right lateral border [15, 16]	
Visual assessment of SAM	PLAX view	2D and M-Mode	Place M-Mode cursor through the MV leaflet tips, ensuring image is on axis. Involves MV leaflets and/or chordae [15, 16]	
Visual assessment of SAM	PSAX view	2D	Parasternal short axis window (PSAX) to demonstrate best image available [15, 16]	
MR secondary to SAM	A4C A3C	2D CF/CW Doppler	MR Quantification may be limited as the PISA dome may merge with turbulent flow. MR secondary to SAM is predominantly posteriorly directed and originates more medial in the apical 4 chamber view. If quantification assessment of MR is precluded by LVOTO, other indicators of MR severity should be considered [17]. For example, an E velocity of < 1.3 m/s and an E/A ratio < 1 are strongly suggestive of non- severe MR [15, 16]. CW Doppler velocity from MR jet facilitates differentiation of LVOT flow from MR in A5C	

LV Simpson’s Biplane volumes	A4C, A2C	2D Units: EDV mL ESV mL	LV volumes should be obtained using 2D imaging from A4C and A2C [18]. Trace the endocardial border. LV length is defined as the distance between the midpoint of the mitral valve level line and the most distal point of the LV apex. Take care to ensure the LV is not foreshortened. Papillary muscles and trabeculations are excluded from the volumes and considered part of the chamber. Measure at end-diastole and end-systole. Measurement is indexed to BSA. Consider LV opacification for improved endocardial definition. 3D assessment of the LV is a highly feasible and reproducible parameter of LV systolic function. Given the superiority over 2D estimates, 3D assessment of LV volumes and LVEF is recommended in cases were image quality permits accurate measurement. Given the additional time involved this is not mandated where accurate LVEF can be provided by Simpson’s biplane assessment. A numerical value for LVEF should be included in all echo reports. A visual estimate should be provided only when image quality is suboptimal Simpson’s biplane estimate [18]	



LVOT gradients at rest/Valsalva manoeuvre	A5C A3C	CW Doppler	For the mechanics of performing a Valsalva manoeuvre, please refer to the BSE HCM protocol [15] Assess LVOT obstruction gradients at rest, with Valsalva manoeuvre. Align CW Doppler through entire turbulent colour flow for peak obstruction velocity [15, 16]. Peak velocity recorded on A5C or A3C should be recorded	

### Optional measurements

**Table:** 

Measurement	View	Modality	Explanation	Image
LV GLS Measurement	A4C A3C A2C	LV GLS	Optimal ECG signal with minimal heart rate variability should be present across the three cardiac cycles. Heart rate variability will limit the circulation of GLS values, which can be problematic in patients with atrial fibrillation. High -quality acquisition, maintaining a frame rate of 40–90 frames/sat a normal heart rate is key [18] Clear endocardial and epicardial definition is required to ensure adequate segmental tracking throughout the cardiac cycle. Markers are placed in each of the respective basal and apical regions, using automatic tracking where possible to maintain reproducible results. Automatic tracking should be combined with visual assessment of tracking in each view across the whole ROI, including the endocardial and epicardial border. If more than two segments in more than one view are not adequately tracked, the calculation of GLS should be avoided [18]	



LA strain	A4C A2C	LA Strain	When acquiring images for LA strain analysis, dedicated atrial windows should be acquired to maximum LA volume. LA strain analysis in views that are optimised for the LV therefore forshortening leads to overestimation of LA values [19]	
TDI velocities	A4C	PW TDI Septal and lateral walls Units: cm/s	Place sample volume (5-10 mm) at or within 1 cm of the insertion of the mitral valve leaflets. Measure at the end of expiration. Scale and sweep speed optimised (100 mm/s) [18]. e’: Peak velocity at the leading edge of spectral wave form in early diastole (after T wave). If possible average both septum and lateral wall measurements S’: Peak systolic Velocity Limitation: this measure assumes that the function of these two segments represents longitudinal function of the entire ventricle, this is unlikely in conditions that result in regional wall motion abnormalities [18]. Place sample volume (1–3 mm) at level of the MV leaflet tips in diastole. Use of CFD can help to align the centre of trans-mitral flow. Measure at end expiration. Emax: peak velocity in early diastole. Amax: peak velocity in late diastole (after P wave). DT: Flow deceleration time from peak E wave to end of E wave signal [18].	

PW doppler mitral inflow	A4C MV (PWD)	EV max AV max E/A ratio DT	Obtain RV focussed view. From A4C view slide and/or angulate the tail of the transducer along the horizontal plane to place the RV in the centre of the image (instead of the conventional left heart-centred image) whilst ensuring that the LV outflow tract does not come into view. This allows the RV free wall to be clearly seen. Next, rotate the transducer to obtain the maximum diameter [18]	
RV focussed view	A4C RV/RA (2D)	Visual assessment	Perform CW Doppler and colour quantification for TR in all views where the TV is visualised. See BSE pulmonary hypertension guidelines for estimating probability of pulmonary hypertension [20]	
	A4C	TR Vmax, extent of TR		
	A4C Lat TV/MV annulus (MM)	TAPSE	Align the M-mode cursor along the direction of the lateral tricuspid or mitral annulus to maximise longitudinal motion of the annulus. Measurement accuracy is improved by zooming on the TV annulus and selecting a high sweep speed. Measure total excursion of the tricuspid annulus [20]. Limitation: This is an angle dependent measurement and is therefore underestimated when M-mode alignment is not parallel [21]. TAPSE is further limited due to the assumption that the longitudinal motion of this single region of the annulus represents the function of the entire RV, this is unlikely in cases of RV regional wall motion abnormalities [21]	
	A4C RV (TDI)	RV S′	PW tissue Doppler S wave measurement taken at the lateral tricuspid annulus in systole. It is important to ensure the basal RV free wall segment and the lateral tricuspid annulus are aligned with the Doppler cursor to avoid velocity underestimation [22]. As well as Doppler alignment limitations, the accuracy of this measurement is further limited by the assumption that overall function of the RV is reflected by basal RV contraction. Accuracy of this measure is therefore limited by conditions such as RV infarction and prior cardiac surgery [23]	
LVOT gradients when standing	A5C A3C	CW doppler	Assess LVOT obstruction gradients when standing. Also assess when standing with Valsalva manoeuvre to maximise velocity observed. Align CW Doppler through entire turbulent colour flow for peak obstruction velocity [15, 16]. Peak velocity recorded on A5C or A3C should be recorded	

### Minimum dataset for TTE for patients with obstructive hypertrophic cardiomyopathy receiving cardiac myosin inhibitor treatment

**Table:** 

View	Parameters
PLAX	2D
	Colour Doppler for MV, LVOT and AV
	M-Mode of MV leaflets for SAM
PSAX	2D MV Level
AP4C	2D and focused for Biplane Simpsons and for GLS (GLS is optional) 2D and focused LA for LA strain (optional)
	Colour Flow Doppler of MV
	CW Doppler for MR
	PW Doppler for Mitral inflow/TDI velocities at LV septal/lateral regions (Optional)
AP5C	2D
	Colour Doppler for LVOT
	CW Doppler for LVOT resting/Valsalva
AP2C	2D and focused for Biplane Simpsons and for GLS (GLS optional) 2D and focused LA for LA strain (optional)
	Colour Doppler for MR
	Optional RV assessment recommended at week 12- 2D, CW doppler of TV, TR CW doppler, TAPSE, TDI, PASP
AP3C	Valsalva with CW for LVOT
	Colour Doppler for MR
	Colour Doppler for LVOT, 2D for GLS (Optional)

### Contrast echocardiography/ultrasound enhancing agents

Suboptimal endocardial border definition can lead to errors in LV volume and LVEF estimation. Accurate LVEF assessment is essential during surveillance of patients being treated with Mavacamten, when values obtained fall on boundaries this may influence treatment decisions. The BSE recommend that contrast is used when two or more segments cannot be visualised [24]. LV volumes in both systole and diastole are greater when measured using contrast agents than without as tracing of the LV borders more reliably leads to inclusion of trabeculation within the cavity. Minimal detectable difference for 2D contrast LVEF has been noted to be in the order of 4% in comparison to 9–11% for non-contrast 2D LVEF [25]. The use of contrast can have unpredictable effects on 2D speckle tracking, therefore it is recommended the use of contrast is given after strain acquisition [26]. If a contrast agent is required, it is recommended the BSE contrast Echocardiography: Practical guideline is adhered to [24].

### Stress echocardiography

Exercise stress echocardiography may be required as part of standard of care for those symptomatic patients without obvious gradients at rest or with Valsalva (gradients < 50 mmHg). Stress Echocardiography using a treadmill or bike is recommended in patients with HCM, dobutamine infusion is not used as this is known to produce increased outflow Doppler velocities in normal hearts. If exercise stress echo is required please refer to the stress protocol within the BSE Hypertrophic Cardiomyopathy guidelines [15, 16]. Stress echocardiography is not recommended as part of routine surveillance for Mavacamten therapy.

## Conclusion

Mavacamten is the first licensed cardiac myosin-inhibitor. Due to the potential for excessive reduction in LVEF [12, 13] surveillance of LV function is mandatory [1]. Echocardiography is a key imaging modality in the initial assessment and subsequent monitoring of patients treated with Mavacamten. High quality LVEF assessment is vital along with accurate LVOT gradient acquisition, including assessment with Valsalva manoeuvre. These assessments are essential and are key when optimising and monitoring safety in patients treated with Mavacamten. Additional key echocardiographic indices can also be assessed at Mavacamten surveillance echocardiography according to local capacity, and routine standard of care echocardiograms adhering to BSE guidelines [15, 16] should continue in parallel with a full data set collected.

## Data Availability

No datasets were generated or analysed during the current study.

## Appendix 1

Prior to initiation of Mavacamten, it is recommended that patients undergo pharmacogenomic testing in order to assess metabolic activity linked to removal of Mavacamten. As Mavacamten is metabolised through the hepatic enzymes CYP2C19 (74%), CYP3A4 (18%) and CYP2C9 (7.65%) genotyping of CYP2C19 is required to guide Mavacamten therapy and dosing. Depending on results, patients will be characterised as rapid, normal, intermediate or poor (slow) metabolisers. Those patients who are “poor” metabolisers have the risk being exposed to higher than intended doses of Mavacamten (due to reduced clearance) therefore adjustments need to be made during dose titration [27]. In the situation where there may be a delay gaining access to CYP status patients may be initiated at a low dose of 2.5 mg until CYP status is known.
